# How Wood Would Build the Canal

**Published:** 1902-10

**Authors:** 


### How Wood Would Build the Canal.

AMERICAN enterprise and modern hygiene embodied in one person — General Wood — startled the world by demonstrating that tropical climate alone is powerless to cause tropical dysentery, cholera, typhoid fever, or yellow fever. This astounding fact seems to be the inevitable conclusion from watching the effect upon a perennial plague spot of enforced cleanliness. The whole world now knows the true relation to health of the climate of Cuba, and also has a better conception than ever before of the pathogenic possibilities of the filthy habits of the Cubans.

Our nation is likely to come again under the observation of the world in a conspicuous way when we build the transisthmian canal at Panama or elsewhere, as may be determined. In the discussions, in print and before congress, upon the possibility or desirability of this canal, the conclusion seems to have been universal that if the task was undertaken it would only be completed at an enormous loss of life to the workers from the diseases incident to the climate—those above mentioned; that white labor could not be employed in the work, or if employed it would be with the understanding of the enormous liability to disease of a high percentage of fatalities. But Wood's sanatory compaign in Cuba has demonstrated the exact reverse of this conclusion, and we have the absolute conviction that under his direction, or that of an equally efficient sanitarian,—if he can be found—the canal can be built from start to finish with immunity to the entire force from the so-called tropical diseases. Further, that the cost of the requisite sanatory equipment, including sanatory camps, hygienic water and food, inspection and maintenance, assuming that 1,000 men may be gathered in a single camp, will not exceed $10.00 a man a year.

By the term sanatory camp, we mean camps upon a site selected by the sanatory officer, with provision made for piping to all of its parts an abundant supply of pure water, with proper sewers in all its parts, and with a generous provision of concrete or other impervious covering for the more exposed portions of its soil—to the end that in that camp men may live without infecting or fouling the soil, may eat clean food, drink clean water, and sleep undisturbed by disease-bearing insects. From such camps men would go to their daily tasks fit for their duties; in them tired men would find rest and refreshment from toil, and immunity from disease. Inspection should show that members of the expedition were sound at the beginning, and daily inspection of camps and men would help to preserve such soundness. What more impressive lesson to the world could be given of the value of modern sanitation than the object lesson of a sanatory outfit building the trans-isthmian canal in defiance of nature and of tropical climate without infection from tropical disease. And who is better fitted for this conspicuous task by training, knowledge and experience than our distinguished and honored confrère General Wood ?

